# Effect of strength-based physical exercise on telomere length as a marker of premature ageing in patients with schizophrenia: study protocol for a pilot randomised controlled trial

**DOI:** 10.1192/bjo.2024.753

**Published:** 2024-09-26

**Authors:** Juan Luis Sánchez-González, Raúl Juárez-Vela, Virginia Dutil Muñoz de la Torre, María del Pilar Andrés-Olivera, Javier Martín-Vallejo, Álvaro Morán-Bayón, Joana Isabel Gonçalves-Cerejeira, Nerea Gestoso-Uzal, Rogelio González-Sarmiento, Jesús Pérez

**Affiliations:** Department of Nursing and Physiotherapy, University of Salamanca, Spain; and Department of Neuroscience, Institute of Biomedical Research of Salamanca (IBSAL), Salamanca, Spain; Faculty of Health Sciences, University of La Rioja, Logroño, Spain; and Department of Neuroscience, Institute of Biomedical Research of Salamanca (IBSAL), Salamanca, Spain; Psychiatry Service, Salamanca Health Care Complex, Salamanca, Spain; Department of Statistics, University of Salamanca, Spain; Department of Medicine, University of Salamanca, Spain; and Department of Neuroscience, Institute of Biomedical Research of Salamanca (IBSAL), Salamanca, Spain; Psychiatry Service, Avila Health Care Complex, Avila, Spain; Department of Molecular Medicine, Institute of Biomedical Research of Salamanca (IBSAL), Salamanca, Spain; Department of Medicine, University of Salamanca, Spain; Department of Neuroscience, Institute of Biomedical Research of Salamanca (IBSAL), Salamanca, Spain; Department of Psychiatry, University of Cambridge, UK; and Norwich Medical School, University of East Anglia, Norwich, UK; Department of Medicine, University of Salamanca, Spain; and Department of Molecular Medicine, Institute of Biomedical Research of Salamanca (IBSAL), Salamanca, Spain

**Keywords:** Ageing, exercise, mortality, schizophrenia, telomere

## Abstract

**Background:**

Patients with schizophrenia die decades earlier than the general population. Among the factors involved in this mortality gap, evidence suggests a telomere length shortening in this clinical population, which is associated with premature ageing. Recent studies support the use of strength-based training exercise programmes to maintain, or even elongate, telomere length in healthy elderly populations. However, studies aiming at modifying telomere length in severe mental illnesses, such as schizophrenia, are still very scarce.

**Aims:**

To investigate the effect of a strength-based physical exercise programme on the telomere length of individuals with schizophrenia.

**Method:**

We propose a pragmatic, randomised controlled trial including 40 patients aged ≥18 years, with a stable diagnosis of schizophrenia, attending the Complejo de Rehabilitación Psicosocial (CRPS, Psychosocial Rehabilitation Centre) in Salamanca, Spain. These patients will be randomly assigned (1:1) to either receive the usual treatment and rehabilitation programmes offered by CRPS (treatment-as-usual group) or these plus twice weekly sessions of an evidence-based, strength-based training exercise programme for 12 weeks (intervention group). The primary outcome will be effect on telomere length. Secondary outcomes will include impact on cognitive function, frailty and quality of life.

**Results:**

We expect to show the importance of implementing strength-based physical exercise programmes for patients with schizophrenia. We could find that such programmes induce biological and genetic changes that may lengthen life expectancy and decrease physical fragility.

**Conclusions:**

We anticipate that our trial findings could contribute to parity of esteem for mental health, reducing premature ageing in patients with severe mental illnesses, such as schizophrenia.

Schizophrenia can have a significant impact not only on mental health, but also on physical well-being.^1–3^ This chronic and debilitating mental disorder is characterised by a blend of hallucinations, delusions, disorganised thoughts, erratic behaviour and negative symptoms such as poverty of speech, lack of motivation or loss of interest in enjoyable activities.^4^ Individuals with schizophrenia experience a higher incidence of physical health problems compared with the general population. This may be attributed to the side-effects of antipsychotic medications, insufficient physical activity and a higher prevalence of unhealthy lifestyle habits such as smoking, illicit drug use and alcohol consumption.^5^ Several studies have shown that people with schizophrenia have a significantly shorter life expectancy than the general population, largely because of higher premature mortality rates.^6,7^ An observational study published in 2017 found that the standardised mortality rate in individuals with schizophrenia was more than twice that of the general population, with an even higher risk for those with early-onset schizophrenia.^8^

This premature mortality could also be linked to accelerated telomere shortening. Telomeres are protective structures at the ends of chromosomes that shorten with each cell division.^9^ Telomere length is associated with cellular ageing and health deterioration. Multiple studies have established that populations with shorter telomeres have an increased risk of chronic diseases and higher mortality rates.^10–12^ Telomere shortening has been related to different factors, including genetic causes. For instance, certain gene polymorphisms involved in the expression of telomerase, an enzyme responsible for maintaining and elongating telomeres, can have an impact on its function and, consequently, on telomere length. In fact, functional single nucleotide polymorphisms (SNPs) in the *TERT* gene can affect telomerase expression and activity, as shown in a meta-analysis conducted by Liu et al.^13^ In addition, SNPs in the loci encoding *TERT* and *TERC* genes are associated with telomere length and also an increased risk of age-related diseases and mortality.^14–17^

A recent systematic review with meta-analysis revealed that people with an established diagnosis of schizophrenia, typically more than 5 years from its onset, had shorter telomeres compared with individuals without mental disorders, indicating premature cellular ageing.^18^ These findings highlight the importance of identifying organic risk factors in vulnerable individuals with schizophrenia, and improving access to long-term physical care and monitoring to prevent premature mortality. Furthermore, these results also suggest that interventions to delay or prevent telomere shortening may be beneficial for those diagnosed with schizophrenia.

One of the non-pharmacological treatments that has demonstrated a positive impact on the physical and mental well-being of individuals with schizophrenia is physical exercise.^19^ Firth et al conducted a systematic review investigating the effects of physical exercise on the mental and physical health of individuals with schizophrenia. They found that physical exercise can ameliorate symptoms of the illness, enhancing quality of life and improving physical functioning.^20^ Many scientific contributions advocate for physical exercise as a first-line non-pharmacological treatment to address physical and cognitive impairments in people with schizophrenia.^20–22^ On the other hand, recent studies have shown that strength-based training exercise has beneficial effects on the telomere length of elderly populations.^23^

However, despite all this evidence, there is no interventional study assessing the impact of physical exercise, specifically strength-based, on telomere length in individuals with schizophrenia, who exhibit shorter telomeres.

## Main and secondary objectives

The main objective of this study will be to investigate the effect of strength-based physical exercise on telomere length in individuals diagnosed with schizophrenia. The secondary objectives will be to:

- explore the impact of physical exercise and telomere length on cognition;
- examine the influence of physical exercise and telomere length on frailty;
- assess the effect of physical exercise and telomere length on the quality of life;
- determine potential correlations between physical parameters related to frailty and telomere length;
- establish the importance of telomerase (enzyme involved in telomere length maintenance) gene polymorphisms.

## Method

We propose a pragmatic, randomised controlled trial, where we shall test the effect of a 12-week strength-based training exercise programme on the telomere length of individuals with schizophrenia. The trial follows the Consolidated Standards of Reporting Trials (CONSORT) Statement^24^ and the treatment protocol is described in accordance with the recommendations of the Standard Protocol Items: Recommendations for Interventional Trials (SPIRIT).^25^ The study will be conducted from 1 January 2024 to 1 September 2024.

The authors assert that all procedures contributing to this work comply with the ethical standards of the relevant national and institutional committees on human experimentation and with the Helsinki Declaration of 1975, as revised in 2008. All procedures involving human patients were approved by the Research Ethics Committee at the Salamanca University Healthcare Complex on July 2023 (reference 2023/07). The trial is registered with Clinicaltrials.gov (registration number NCT05978921).

All patients in the study will be informed about the objective of the study and will give their written informed consent to participate in the study. The patients’ right to privacy will be respected, the applicable data protection laws in force will be observed and the anonymity of all study participants will be guaranteed when data are presented in scientific journals. Patients’ medical data will be considered confidential, and disclosure to third parties will be forbidden. To ensure proper confidentiality, identification numbers will be randomly assigned to each participant. The intervention sessions will be adapted to each patient, making those modifications in the intervention that are necessary to maintain patient's safety. The risk of therapy-related adverse events is minimal. However, if (serious) adverse events should occur during or after the study, these will be monitored by the study team and physicians as part of standard therapy. All serious adverse events will be documented and reported immediately (within a maximum of 24 h) to the principal investigators of the study.

### Study participants and setting

Participation in the study will be voluntary, and potential participants will be provided with explanations of the study objectives and methodology before completing and signing an informed consent form.

#### Inclusion criteria

Inclusion criteria are patients with an established DSM-5-TR diagnosis of schizophrenia for at least 5 years, who are attending the Complejo de Rehabilitación Psicosocial ((CRPS) Psychosocial Rehabilitation Centre) or its associated out-patient clinic for severe mental disorders in Salamanca, Spain. The CRPS is an out-patient rehabilitation centre that, in addition to pharmacological treatment and follow-up by mental health professionals, offers a series of rehabilitation programmes in the community, including psychosocial interventions, social reinsertion, employment advocacy and support, and family interventions, among others.

#### Exclusion criteria

Exclusion criteria are patients without mental capacity to understand the study information sheet or to sign the informed consent. Also, those who would be unable to engage in a physical training of a moderate intensity because of medical reasons will be excluded from the study.

### Study intervention

Participants will be randomly assigned to either the intervention or treatment-as-usual groups.

Participants in the intervention group will have access to the usual treatment programmes at CRPS, but will also enrol into twice weekly 50 min sessions of strength-based physical exercise over 12 weeks. Patients in the treatment-as-usual group will continue accessing the traditional CRPS programmes, but will not participate in the strength-based exercise sessions.

The exercise sessions will be carried out at the ‘Contemporánea’ Physiotherapy Clinic in Salamanca (https://www.fisiocontemporanea.com/).

In accordance with previous studies involving moderate exercise training programmes for patients with schizophrenia, the duration of the intervention will be limited to 12 weeks.^26,27^ The training sessions will follow an evidence-based physical activity programme with successful outcomes on telomere length in healthy populations,^23^ and will be structured as follows:

- Part 1: Warm-up with an aerobic component lasting approximately 15 min.
- Part 2: Twelve multi-analytical exercises based on the strength endurance component. Six of these exercises will be performed during the first weekly session, and the rest during the second. Each exercise will comprise three sets of 12 repetitions. The proposed exercises are as follows:
  - First session: squat and row with band; glute bridge and kettlebell chest press; backward lunge and abdominal pull-off with band; push up; glute kickback with band; abdominal crunch.
  - Second session: wall ball squat; step up and kettlebell overhead press; abdominal pull-off and lateral split squat with slider; pullover with band; kettlebell deadlift; kettlebell split squat.
- Part 3: Cool-down phase with breathing and stretching exercises.

Before the intervention begins, participants will undergo two to three preparation sessions, in which they will practice the exercises that they will later carry out over the 12 weeks.

These sessions will also help tailor the exercise load for each individual. Participants will be introduced to the Rating Scale of Perceived Exertion (RPE),^28^ based on the number of repetitions in reserve (RIR).^29,30^ Participants will be encouraged to perceive an effort level between 7 and 8 on the RPE scale^28^ (0–10; 0 being no effort at all and 10 being maximal effort) during each exercise. If participants consistently complete 12 repetitions with lower perceived exertion than specified in two consecutive sessions, the training load will be increased by approximately 2–10%, following the guidelines of the American College of Sports Medicine.^31^ The RIR-based RPE will be re-evaluated.

### Patient and public involvement

Patients have been involved in the design and will participate in the delivery of this trial. For instance, patients with schizophrenia currently attending the CRPS were consulted on the feasibility of running this project and some of them were involved in its design, specifically on where and how to carry out the training sessions. In addition, two CRPS staff members, with lived experience of severe mental illness, may support physiotherapists at the ‘Contemporánea’ Physiotherapy Clinic in delivering physical training sessions (the intervention).

### Variable and outcomes: type and measurement

#### Primary variable and outcome

##### Telomere length

Telomere length will be calculated using a protocol similar to other studies.^23^ DNA will be extracted from saliva following a specific protocol.^32,33^ Cells will be isolated through centrifugation and resuspended in Fornace buffer. The resulting pellet will undergo incubation for protein degradation and cell membrane breakdown. Subsequently, DNA will be extracted, purified using the phenol-chloroform method and precipitated with cold absolute ethanol.

The concentration of the extracted DNA will be determined by measuring absorbance at 260 nm, using a NanoDrop 2000/2001 spectrophotometer. DNA purity will be assessed based on the A260/280 absorbance ratio, with an optimal ratio ranging from 1.8 to 2.0. Extracted DNA samples will be stored in Eppendorf tubes at −20 °C.

Telomere length in saliva cells from each participant will be measured with quantitative real-time polymerase chain reaction in conjunction with the Absolute Human Telomere Length Quantification qPCR Assay Kit (ScienCell, Catalog #8918, California, USA). This technique quantifies the initial amount of DNA coding for telomerase (TEL) and compares it with that from another fragment corresponding to a single-copy reference gene (SCR) for endogenous control. The difference in the amount of DNA quantified represents each participant's relative telomere length. A reference fragment (CONTROL) with a known telomere length, provided by the manufacturer, will be added to each assay, to allow absolute quantification of telomere length in each sample.

The reactions will occur in a Micro-Amp Fast Optical 96-Well reaction plate (Applied Biosystems), using the Applied Biosystems StepOnePlus Real-Time PCR System. Triplicate reactions will be performed for each sample, to minimise variability. The amplification program will consist of 10 min at 95 °C, followed by 40 cycles at 95 °C for 15 s, 52 °C for 30 s and 60 °C for 1 min.

The Ct (2^−ΔΔCt^) comparative method will be employed to calculate the relative expression levels of each amplicon. The specificity of each polymerase chain reaction will be confirmed by verifying the telomere length of the reference sample (CONTROL), which, in turn, allows determination of absolute lengths for each sample for a diploid cell and/or chromosome end. Data management will be conducted with Microsoft Excel, version 16 for Windows.

#### Secondary variables and outcomes

##### Cognitive function

Cognition will be assessed with the Brief Assessment of Cognition in Schizophrenia (BACS).^34^ The BACS score ranges from 0 to 100 points, with a higher score indicating better cognitive performance within the normative population.

##### Frailty

Frailty will be measured with the Short Physical Performance Battery scale (SPPB),^35^ which comprises three components: a balance test, walking time and standing from a seated position. The maximum score is 12 points, with a higher score indicating better physical functioning.

##### Quality of life

Quality of life will be evaluated with the EuroQuality of Life (EQ-5D-5L scale),^36,37^ which ranges from 0 to 100, with 100 representing the best imaginable health and 0 representing the worst.

In addition to the primary and secondary variables, we shall collect basic sociodemographic information, such as age (years), gender (female), weight (kilograms) and height (centimetres).

### Sample size

Because of the lack of previous interventional studies looking into changes in telomere length following physical exercise in patients with schizophrenia, the sample size calculation was based on a pre–post study, using the proposed intervention in elderly healthy participants.^23^ Accordingly, for an identified Cohen's effect size of 1.1, a statistical power of 90% and an alpha level of 0.05 with a one-sided contrast, we would need a sample size of 32 individuals (16 per group). To account for potential study losses, a 20% lost-to-follow-up rate was considered, leading to a required sample of 40 participants (20 per trial arm). We employed the R Statistical Software pwr package for this calculation (version 4.4.2 for Windows, The R Foundation; https://www.r-project.org/).

### Randomisation

Participants who meet the inclusion/exclusion criteria and want to participate in the study will be randomly assigned to either the treatment-as-usual group or the intervention group (treatment-as-usual plus a strength-based training programme.) We shall carry out a permutation block randomisation (1:1) by generating a set of random numbers where the even numbers define a block of specified ordered randomly treatments assignment and the odd numbers define other order of assignment of treatments. The random permuted blocks method should achieve balanced experimental groups.

### Data analysis

We shall describe data emerging from the study by using means and s.d., and medians and interquartile ranges for quantitative variables, and percentages for qualitative values. Normality will be assessed with the Kolmogorov–Smirnov test with the Lilliefors correction, and by examining box plots.

For group comparison, we shall employ the *t*-test for independent variables when these follow a normal distribution, and non-parametric Mann–Whitney *U-*test for non-normal distributions. The Levene test will be used to assess population equality variances. To analyse changes over time for unidimensional measurement tools within the trial groups, a two-factor analysis of variance will be used, with one factor being independent variables (intervention and treatment-as-usual group) and the other dependent (pre, post). If the tool has multiple dimensions, a three-factor analysis of variance will be used, with one independent factor (intervention, control) and two dependents (time: pre–post and dimension). In the event that there is missing data, we will use mixed models. We shall also use *post hoc* Bonferroni corrections.

All parameters will be presented with 95% confidence intervals, and statistical significance will be determined when *P*-values are <0.05. Data analyses will be performed with IBM SPSS version 28 for Windows and R statistical software.

### Task distribution

All authors will have a direct participation in the development of this clinical trial.

The recruitment, delivery and collection of informed consent will involve several team members: J.P., M.P.A.-O., A.M.-B., V.D.M.T. and J.I.G.-C. Once this step is completed, randomisation will be carried out by J.M.-V. and R.J.-V. Initial assessments and data collection will be divided into two main areas, clinical and genetic. J.L.S.-G., V.D.M.T., M.P.A.-O., A.M.-B. and J.P. will perform clinical evaluations, and N.G.-U. and R.G.-S. will be responsible for the analysis and calculation of telomere size. Preparation of training sessions will fall under the responsibility of J.L.S.-G., who will organise and coordinate them. Strength-based training exercise sessions will be conducted by physiotherapists at the Contemporánea Physiotherapy Clinic, with the support of CRPS employees with lived experience of psychotic disorders. Follow-up data collection will be carried out by J.L.S.-G., V.D.M.T., M.P.A.-O., A.M.-B., J.P., N.G.-U. and R.G.-S. Finally, the analysis and writing of the results will be shared among J.P., J.L.S.-G., J.I.G.-C., R.J.-V., J.M.-V. and R.G.-S.

## Discussion

There are many studies that have evaluated the impact of physical exercise on different clinical domains in schizophrenia, such as cognition,^38^ physical health^39^ and functioning.^40^ However, the effect of physical exercise on telomere size in people with schizophrenia has not yet been investigated. Here, we present the protocol of a pragmatic, randomised controlled trial to address this important scientific shortage, aiming to address one of the greatest healthcare challenges worldwide: the mortality gap between people with severe mental illnesses, such as schizophrenia, and the general population.

### Strengths

With this study we expect to show the importance of implementing strength-based physical exercise programmes for people with schizophrenia. We could find that such programmes induce biological and genetic changes that contribute to lengthen the life expectancy and decrease the physical fragility of a highly vulnerable population that die decades before their time. Also, the novelty of this work and its goal, which is highly prioritised in public health and health services development strategic agendas in different countries, such as the UK,^41^ suggest that a positive result might be considered in future, updated health policies and treatment guidelines for patients with schizophrenia. This is the first clinical trial that will assess the impact of strength-based physical exercise on telomere length in individuals with schizophrenia as a marker of premature ageing. The primary outcome (effect on telomere length) will be objectively measured by validated laboratory techniques. This will provide a strong scientific basis for data interpretation.

We anticipate that our trial findings might represent a significant leap toward parity of esteem between the healthcare and research that non-psychiatric patients with chronic diseases receive (or have access to) to increase their life expectancy, and those for patients with severe mental disorders, who are at a high risk of chronic organic diseases that are often overshadowed by mental health issues – a fact that leads to premature death.

### Limitations

Our study has some limitations mainly related to its pragmatic nature. For example, trial participants´ physical activities outside of the healthcare system will not be controlled, and there will not be a healthy volunteer group to make comparisons. Nonetheless, our study population tends to live a more passive life in terms of physical exercise than the general public; thus, it is unlikely that this would affect the trial findings. To further temper this limitation, all trial participants will be administered the International Physical Activity Questionnaire to monitor their pre-trial physical activity levels. Also, there is evidence suggesting that telomere length may vary depending on whether samples are obtained from saliva or peripheral blood.^42^ Nevertheless, we shall use saliva samples in all our measurements, which will avoid potential bias related to the DNA extraction methodology. Furthermore, telomere length may be affected by many factors, such as smoking tobacco, alcohol consumption and socioeconomic deprivation. We will take this information into consideration for the analysis and interpretation of the results; however, since the study does not entail any other change in the patients´ life than a possible allocation to a strength-based training exercise programme, if this eventually had an effect, it would still be reflected in intra-individual telomere length changes. The study will be carried out in only one site and will include a relatively low number of participants, which may affect the generalisability of the results and, therefore, it may need to be replicated elsewhere. Given the pragmatic nature of this trial, other factors that may affect telomere length, such as smoking tobacco, will not be controlled.

## Data Availability

Data that support the findings of this study will be made available from the corresponding author, J.P., upon reasonable request after publication of findings.
